# Assessment of Healthcare Providers' Knowledge on the Management of Pediatric Tracheostomy Emergencies

**DOI:** 10.1055/s-0044-1791258

**Published:** 2025-04-28

**Authors:** Maha Alharbi, Rakan Almutairi, Salman Alotaibi, Mohammed Alqarny, Faisal AlMudhaibery, Hussein Etwadi, Yousef Aljathlany, Ahmed AlKhateeb, Abdulaziz AlEnazi

**Affiliations:** 1 Department of Otolaryngology – Head and Neck Surgery, Eastern Health Cluster, Dammam, Saudi Arabia; 2 Department of Cardiology, Second Health Cluster, Riyadh, Saudi Arabia; 3 Department of Otolaryngology, College of Medicine, Shaqra University, Riyadh, Saudi Arabia; 4 Department of Otolaryngology – Head and Neck Surgery, University of Bisha, Bisha, Asir, Saudi Arabia; 5 Department of Radiology, King Abdulaziz Medical City, Riyadh, Saudi Arabia; 6 Department of Otolaryngology – Head and Neck Surgery, Maternity and Children Hospital, Abha, Saudi Arabia; 7 Department of Otolaryngology Head & Neck Surgery, Imam Abdul Rahman Bin Faisal University, Saudi Arabia; 8 Department of Otolaryngology – Head and Neck Surgery, Security Forces Hospital, Riyadh, Saudi Arabia

**Keywords:** tracheostomy, pediatric, complications, airway, decannulation, knowledge

## Abstract

**Introduction** Morbidity and mortality related to tracheostomy can be reduced if complications are recognized immediately and managed effectively. Healthcare providers involved in the management of pediatric patients must be aware of tracheostomy-related emergencies, especially in life threatening situations. However, there is limited literature on this theme.

**Objectives** To assess the knowledge of healthcare providers in managing tracheostomy-related complications in pediatric patients.

**Methods** A descriptive, multicenter study was conducted among healthcare providers involved in the management of pediatric patients undergoing tracheostomy. Data were collected using electronic questionnaires, and participants' knowledge was evaluated using various clinical scenarios.

**Results** A total of 520 healthcare providers from different subspecialties with varying levels of experience were included. Over 50% of participants had never dealt with tracheostomy-related complications in pediatric populations, and 30.5% were unfamiliar with the function of the introducer/obturator. Furthermore, only 17.9% understood the purpose of stay sutures. Notably, when presented with different clinical scenarios, a significant knowledge gap was observed among the participants.

**Conclusion** There were significant gaps in knowledge among healthcare professionals regarding the management of tracheostomy-related emergencies in the pediatric population. Education on this topic is essential among healthcare professionals. This needs to be addressed to maximize patient safety. Further studies are recommended.

## Introduction

Over the past few decades, tracheostomy has been increasingly performed in children with various complex and chronic medical issues due to advancements in medical care. 1 Currently, the most common indications for pediatric tracheostomy include upper airway obstruction due to structural airway abnormalities, followed by prolonged mechanical ventilation and pulmonary toileting. 1

Pediatric tracheostomy carries significant morbidity and mortality compared with its adult counterpart. According to a recent systemic review, almost 40% of pediatric patients undergoing tracheostomy experience complications, 2 ranging from mild (requiring no intervention) to severe (life-threatening). This group has a higher risk of mortality than adults. However, most of the complications are secondary to patients' comorbidities rather than the tracheostomy itself. 3 The rate of tracheotomy-related mortality ranges from 3.6 to 6%, 2 3 being commonly related to loss of airway caused by tube obstruction, followed by tube misplacement and accidental decannulation. 3

Studies have shown that tracheostomy patients who are transferred from the intensive care unit (ICU) to general wards may receive inadequate care and face serious health risks, as the healthcare providers involved may lack the necessary skills. 4 5 Furthermore, studies have shown that nonsurgical graduate medical education is lacking on this subject. 6 Survey studies conducted among internal medicine, intensive care, and emergency medicine residents showed low levels of comfort and limited training in airway assessment. 6 7

The availability of in-house on-call ear, nose, and throat (ENT) physicians may vary among hospitals. Additionally, over 40% of otolaryngology referrals exceed a waiting time of 3 hours. 8 Considering the urgent nature of tracheostomy complications, such prolonged delays could lead to patient harm or even death when nonotolaryngologist cannot intervene promptly. 6 Therefore, healthcare providers involved in the management of tracheostomized pediatric patients should be aware of potential complications to better manage urgent complications effectively.

The objective of this study was to assess healthcare providers' knowledge about managing emergency complications related to pediatric tracheostomy. To the best of our knowledge, the literature is sparse on this topic.

## Methods

A descriptive, cross-sectional study was conducted using an electronic questionnaire from April to August 2019.

Participants were fully informed regarding the nature and objectives of the questionnaire. The confidentiality and anonymity of our participants were maintained. Ethical clearance and approval were obtained from the institutional Human Research Ethics Committee.

A sample of healthcare professionals ( *n* = 520) from different hospitals and regions in the Kingdom of Saudi Arabia was used. Otorhinolaryngologists, anesthesiologists, pediatricians, intensivists, emergency physicians, and general physicians were included, as well as nurses and respiratory therapists.

The questionnaire was developed following an extensive review of the literature on similar objects by two pediatric otolaryngologist and reviewed by three general otolaryngologists, to ensure each question is clear, concise, and focused. A pilot was tested on a small group at different levels and from different departments before distribution to identify any ambiguities, confusing language, or issues with clarity. At this stage, only minimal changes were made.

The questionnaire was structured into two main sections. In the first one, we collected demographic information such as the primary unit of practice, years of experience, specialization, workplace type, familiarity with tracheostomy procedures, and experience in dealing with pediatric tracheostomy complications.

The second section focused on assessing participants' knowledge about various aspects of tracheostomy, including the most common complications, appropriate actions to take in the event of tracheostomy dislodgment in a newly formed stoma or in difficult intubations. Also, how to address sudden, massive tracheostomy bleeding, as well as knowledge of stay sutures and the function of the introducer/obturator.

Additionally, participants were asked to rate their confidence in inserting a tracheostomy tube in pediatric patients on a scale from 1 to 10 (where 1 means “not confident at all” and 10 “very confident”). We also asked questions about performing a fiberoptic scope exam or neck and chest X-ray after tracheostomy tube change (see Supplementary Material S1 ).

Data management and analysis after collection. Raw data were checked, cleaned, edited, and analyzed using IBM SPSS Statistics for Windows (IBM Corp., Armonk, NY, United States) software, version 25.0. The means, percentages, and standard deviation (SD) values were calculated to describe the profile of the respondents. The Chi-squared test was used to evaluate the statistical significance of the two categorical variables. The one-way analysis of variance (ANOVA) was used to determine the significance of continuous and categorical variables. Values of *p* < 0.05 were considered significant.

## Results

### Demographics and Experience

The questionnaire was sent to a total of 1,502 participants, only 520 participants responded, with a response rate of 34.6%. They represented different specialties. Among them, 262 (50.4%) were male and 258 (49.6%) were female. In terms of hospital affiliation, 380 (73.1%) worked in tertiary, 64 (12.3%) in secondary, and 66 (12.6%) in primary hospitals, as well as 10 (1.9%) in the private sector ( Table 1 ).

**Table 1:** Participant characteristics and experiences

		Total (n = 520)	Percentages
**Gender**	Male	262	50.38
	Female	258	49.62
**Hospital**	Tertiary	380	73.08
	Secondary	64	12.31
	Primary	66	12.69
	Private sector	10	1.92
**Specialty**	ENT physician	64	12.31
	ER physician	49	9.42
	Anesthesia physician	13	2.50
	PICU physician	24	4.62
	Pediatric physician	69	13.27
	Respiratory therapist	65	12.50
	Speech pathologist	12	2.31
	PICU nurse	101	19.42
	ENT nurse	31	5.96
	Pediatric nurse	35	6.73
	ER nurse	17	3.27
	General physician	37	7.12
	Others	3	0.58
**Experience in pediatrics tracheostomy complications**	Yes	240	46.15
	No	280	53.85
**Experience with tracheostomy practice**	< 5 years	208	40.00
	> 5 years	212	40.77
	Other	100	19.23

**Abbreviations:** ENT, ears, nose and throat; ER, Emergency Room; PICU, Pediatric Intensive Care Unit.

In terms of pediatric tracheostomy experience, 208 (40%) participants had less than 5 years of experience, 212 (40.7%) had more than 5 years, and 100 (19.2%) stated that they did not have any experience. Of them, 46.1% reported experience with pediatric tracheostomy complications, whereas 53.8% had none ( Table 1 ).

Responses related to tracheostomy practice were then analyzed with participants' demographics to test statistically significant differences. It was found that consultant and registrar were significantly higher compared with others who had dealt with tracheostomy complications ( *p* = 0.0001). Furthermore, primary health care doctors and those who were working in the medical ward had significantly less experience in dealing with tracheostomy complications ( *p* = 0.009 and 0.0001 respectively).

### Knowledge of Pediatric Tracheostomy Complications

Participants were asked about the most common complications of pediatric tracheostomy. The results of the answers are in ( Table 2 ). Up to 48% of participants answered bleeding as a common complication of pediatric tracheostomy, with 31.5% choosing mucus plug, and 23.4% cannula obstruction.

**Table 2:** Participants' knowledge of most common complication

Complications	Total (n = 520)	Percentage
Bleeding	252	48.4
Mucus plug	164	31.5
Cannula obstruction	122	23.46
Accidental cannula loss	63	12.11
Tracheal spasm	53	10.19
Bronchopneumonia	42	8.07
Aspiration	42	8.07
Pneumothorax	42	8.07
False course of the cannula	37	7.11
Pneumomediastinum	24	4.61

### Knowledge of the Stay Sutures and Introducer

Of the 520 participants, 333 (64%) knew about stay sutures, and the remaining 187 (36%) did not. However, only 17.9% ( *n* = 93) responded correctly when they were asked about the purpose function of the stay suture. The ENT physicians were significantly higher in proportion compared with others who knew the concept of stay sutures ( *p* = 0.0001). When participants were asked about the function of the introducer/obturator, 30.5% ( *n* = 159) stated that they did not know. General physicians (64.8%), Emergency Room (ER) physicians (58.4%), and pediatricians (50%) had higher ratios for this answer compared with others.

### Confidence Level for Inserting the Tracheostomy Tube

Participants were asked to rate their confidence in inserting a tracheostomy tube in case of tube dislodgment ( Table 3 ). The average score for reinsertion was (4.58 ± 3.7). Notably, nurses working in the Pediatric Intensive Care Unit (PICU) and ER, as well as general physicians, expressed lower confidence levels in reinserting the tracheostomy tube. Conversely, respiratory therapists and ENT physicians demonstrated the highest average confidence scores. Additionally, those who had dealt with tracheostomy complications in the past were more confident to reinsert the tube (5.43 ± 3.7) when compared with those without experience (3.42 ± 3.4), the difference was statistically significant, with a *p* -value of 0.0001. Experience was also significantly associated with confidence in reinserting the tube ( *p* = 0.0001).

**Table 3:** Confidence level to insert the tracheostomy tube

Specialty	Score: mean	Score: standard deviation
Respiratory therapist	8.09	2.34
ENT physician	8.06	1.62
ENT nurse	5.41	4.02
Pediatric nurse	4.25	3.45
Anesthesia physician	4.14	3.90
PICU physician	3.79	3.84
ER physician	3.68	3.41
Pediatric physician	3.65	3.54
General physician	2.84	3.44
ER nurse	2.78	2.37
PICU nurse	2.65	2.75

**Abbreviations:** ENT, ears, nose and throat; ER, Emergency Room; PICU, Pediatric Intensive Care Unit.

### Pediatric Tracheostomy Complications Clinical Scenarios

To assess the participants' knowledge of managing challenging complications in pediatric patients with tracheostomy tubes, three distinct clinical scenarios were presented.

**Fig. 1:**
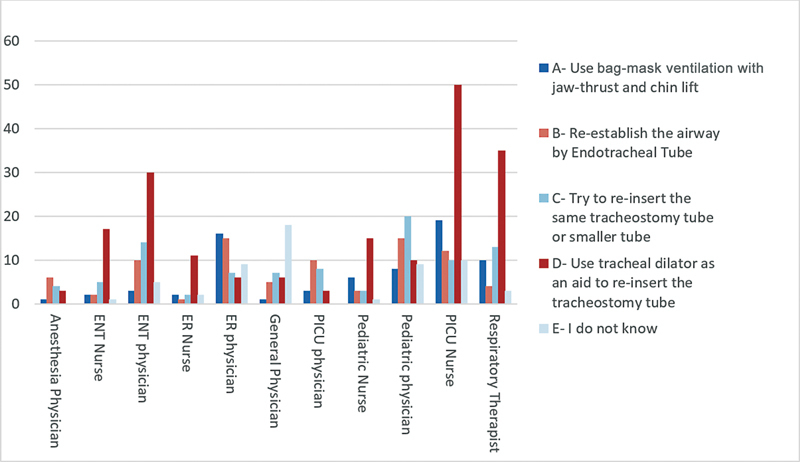
An Intensive Care Unit (ICU) pediatric patient, on day 2 after tracheostomy without stay sutures, got forceful cough that led to accidental dislodgement of tracheostomy tube. What is the first thing you do in such situation?

Clinical scenario 1: “ICU pediatric patient on postoperative day 2 without stay sutures developed a forceful cough that led to accidental dislodgement of the tracheostomy tube. What is the first thing you will do in such a situation?” The specialty-wise responses are presented in Fig. 1 . Most ENT physicians answered that they would use the same tracheostomy tube or a smaller tube (18%) as well as use a tracheal dilator as an aid to re-insert the tracheostomy tube (62%). While anesthesia (42%) and PICU (41.6%) physicians prefer to reestablish the airway by endotracheal tube. Furthermore, pediatric physicians (48.3%), as well as PICU (59.4%), ENT (81%), and ER (72%) nurses would choose the same as the ENT physician.

**Fig. 2:**
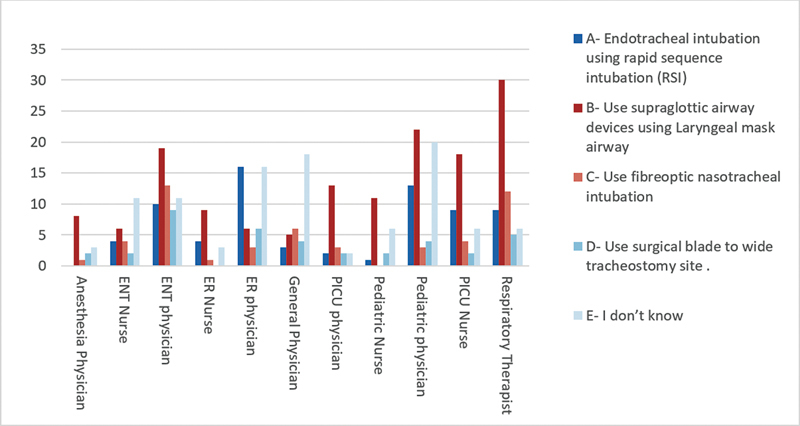
In cases where dislodgement of tracheostomy tube happened and recanulation is impossible, at the same time that intubation is difficult, what can you do to secure the airway in an emergency sitting?

Clinical scenario 2: “How to secure the airway in an emergency setting when dislodgement of tracheostomy tube if recannulation is impossible and at the same time intubation is difficult.” Most of the anesthesia (57%) and PICU (59%) physicians prefer to use supraglottic airway devices using the laryngeal mask airway. The specialty-wise responses are presented in ( Fig. 2 ).

**Fig. 3:**
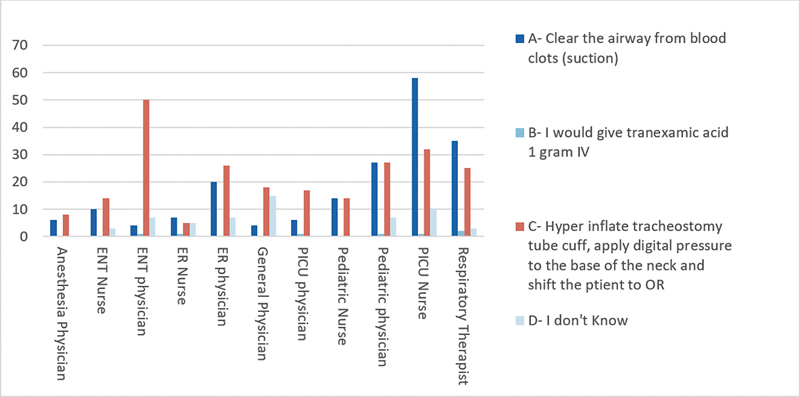
In case of sudden massive tracheostomy bleeding, what you will do?

Clinical scenario 3: “In case of sudden massive tracheostomy bleeding, what will you do?” The results showed that 46.9% answered correctly with a hyperinflate tracheostomy tube cuff, followed by pressuring the base of the neck digitally, and moving the patient to the operating room. The specialty-wise responses are presented in ( Fig. 3 ).

### Use of a Fiberoptic Scope and Neck and Chest X-ray after Difficult Tube Insertion

**Fig. 4:**
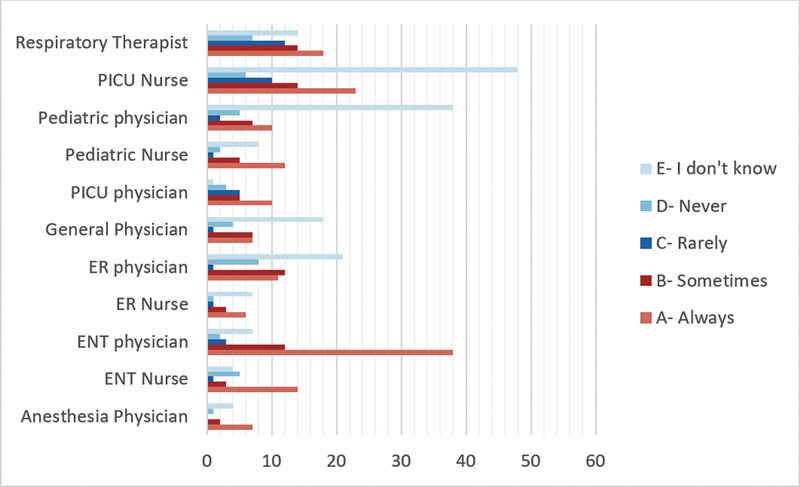
Would you use a fiberoptic scope to examine the airway after a difficult insertion of the tracheostomy tube?

**Fig. 5:**
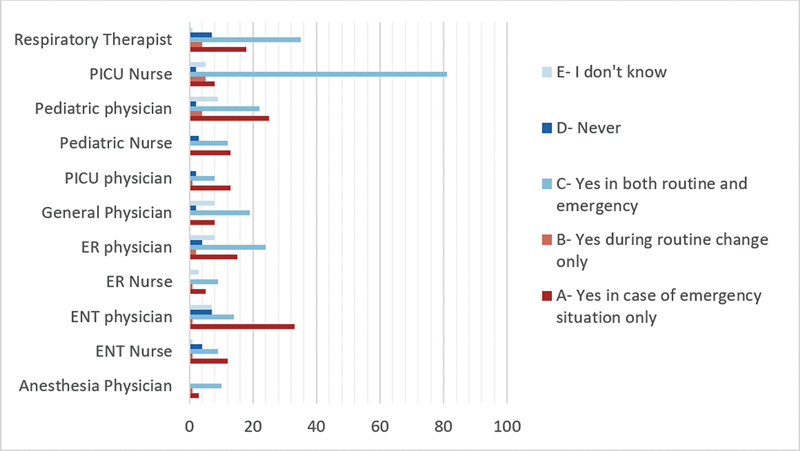
Do you think it is important to request a neck and chest x-ray after tracheostomy changes?

For the assessment of the tracheostomy tube position after difficult tracheostomy tube insertion, ENT physicians (61.3%) and nurses (51.9%), as well as anesthesia physicians (50%), demonstrated a strong preference to “Always” use fiberoptic scopes after difficult tracheostomy tube insertions for the assessment of airway and tube position ( Fig. 4 ). While the majority of specialties acknowledged the importance of neck and chest X-rays, variations in practices were observed. Furthermore, ICU (54%) and ENT (53%) physicians emphasized the need for X-rays, but only in emergencies. In contrast, anesthesia (71.4%) and ER (45%) physicians preferred X-rays during both routine and emergency changes ( Fig. 5 ).

## Discussion

Pediatric tracheostomy is a frequently performed procedure that serves diverse purposes, ranging from addressing neurological complications to managing mechanical airway obstructions. Irrespective of the underlying cause, numerous complications have been documented, with prevalence rates exhibiting significant variations across different studies. A systematic review assessed the incidence of tracheostomy-related complications in the pediatric population and found that the overall rate was 40%. The most common complications were skin lesions, tracheocutaneous fistula, accidental decannulation, and cannula obstruction. 2 They also found that the mortality rate associated with the procedure could reach as high as 6% in pediatric cases and was primarily attributed to issues such as cannula obstruction or accidental decannulation. 2

Our results showed that more than 50% of healthcare providers have never dealt with tracheostomy-related complications in pediatric populations. Furthermore, 21% have never dealt with tracheostomy in pediatric patients, which reflects unequal exposure among different specialties. Most of health care providers in our results showed low level of confidence in reinserting the tracheostomy tube, with the exception of otolaryngologist and respiratory therapists. Notably, participants with prior experience in managing tracheostomy complications displayed a higher level of confidence in reinserting the tube (5.43 ± 3.7) than those without such experience (3.42 ± 3.4). Agarwal et al. assessed the knowledge and confidence levels in tracheostomy care at a tertiary children's hospital among healthcare providers, including residents and hospitalist faculty physicians, self-assessments and objective tests were administered to the participants, which showed a deficiency in their knowledge and confidence in managing routine and emergency tracheostomy care. 9

Early accidental tube dislodgement before tract formation can occur in the postoperative period in either PICU or the pediatric ward. This complication usually requires immediate management to reestablish the airway. However, as the tract is still not mature, reinsertion of the tube can result in false tract passage, which can further aggravate patients' symptoms and increase the risk of bleeding, infection, subcutaneous emphysema, and even cerebral hypoxia, especially if performed by a physician who's unfamiliar with the procedure and its complications. 10 11

To assess the knowledge among health care providers, we gave a scenario regarding a child's 2 ^nd^ day posttracheostomy, with accidental decannulation in PICU without a stay suture. Our results showed that physicians working in PICU, pediatric words, and anesthesia were more comfortable to reestablish the airway using endotracheal intubation and bag valve mask use instead of reinsertion of the tracheostomy tube, which is considered safe, particularly in patients with obesity, and/or short and abnormal neck anatomy. In contrast, ENT physicians were more comfortable reinserting the tracheostomy tube with the help of a dilator, introducer, and stay suture, as they are familiar with the procedure. On the other hand, nurses exhibited varying responses, with those in the PICU or ENT wards leaning toward reinserting the tracheostomy tube, whereas nurses in pediatric wards or ER settings favored bag-mask ventilation and reintubation.

Casserly et al. evaluated healthcare professionals' knowledge of life-saving strategies for patients with tracheostomy by presenting a similar scenario to participants. Their findings showed that 74% of ICU and 100% of ENT nurses believed that reinserting the tracheostomy tube was the appropriate action in such a scenario. 12 The authors concluded that this demonstrates a lack of knowledge among these groups regarding the potential risks of attempted reinsertion, and suggests that attempts among these subgroups are more likely to lead to an adverse outcome. 12 A cross-sectional analysis of survey data from inpatient nursing staff showed that nurses with 5 or more years of experience were more comfortable in managing established tracheostomy tubes than those with less experience. 13 However, only 4% of respondents felt equally comfortable managing an accidental decannulation in a patient with a fresh tracheostomy. 13

Stay sutures are frequently employed in pediatric tracheostomy to facilitate tracheal identification in cases of accidental decannulation of a newly formed tracheostomy. Approximately 94% of pediatric otolaryngologists use it. 14 Healthcare providers must be aware of their presence during accidental decannulation. Our results showed that only 18% of all participants accurately understood the primary function of stay sutures in emergency settings. Furthermore, 30% were not aware of the introducer/obturator. Casserly et al. evaluated healthcare providers' knowledge of stay sutures and found that 23% of the anesthetic group, 37% of the ICU nurses, and 31% of the ENT ward nurses were aware of this procedure's function. 12

During the present study, participants were also presented with a scenario involving a child with accidental decannulation, and recannulation is impossible, at the same time, reintubation is difficult. Our aim was to gain insights into their knowledge and management strategies when faced with difficult intubations. Our results showed that supraglottic airway devices using the laryngeal mask airway is the preferable method among anesthesia and PICU physicians. In an emergency setting, when the tracheostomy tube is dislodged and recannulation is impossible, securing the airway becomes a critical challenge. Healthcare providers should adhere to the guidelines for managing difficult airways in pediatric populations. 15 In cases where intubation is difficult, alternative airway management techniques should be considered; for example, the use of a supraglottic airway device. 15 It is important to note that managing a dislodged tracheostomy tube in an emergency setting can be challenging and require a team approach. The involvement of an airway team, including otolaryngologists, anesthesiologists, and critical care specialists, can help ensure the best possible outcome. 10 15

The occurrence of tracheoinnominate fistula (TIF) in the artery among pediatric patients is relatively uncommon. However, it requires prompt recognition and management because of its potential for catastrophic consequences. More than half of the participants did not know the first immediate management when recognizing TIF. A national survey was conducted among members of the American Academy of Otolaryngology – Head and Neck Surgery to gather qualitative information about catastrophic complications during and following tracheotomy. The survey revealed that pediatric otolaryngologists had twice as many innominate artery fistulas per year of practice compared with others. 16 Furthermore, manifestation of TIF can be more likely in children, which can be attributed to several factors. Pediatric tracheotomy tubes occupy a relatively larger percentage of the airway than in adults, and the pediatric trachea is known to be softer and thinner. 16 17 Additionally, this group is more prone to having an abnormally high-positioned innominate artery, which further predisposes them to the development of TIF. 16 18

Our results showed variations in practices among different specialties in using neck and chest x-rays or fiberoptic scope after difficult tracheostomy tube changes. Despite these variations, there is some consensus among healthcare professionals regarding their application for assessing tracheostomy tube position.

Data regarding neck and chest x-rays or fiberoptic scope after a difficult tracheostomy tube change are sparse in the literature. However, postoperative chest x-rays are a common practice in most healthcare facilities for pediatric patients following tracheostomy. This is mainly due to the perceived higher risk of morbidity and mortality associated with this procedure in children. 19 According to Dane et al., the rate of postoperative complications is surprisingly low, at only 0.71%. Based on their findings, they suggest a selective approach to postoperative chest X-rays in pediatric tracheostomy cases, taking into consideration the low complication rate, cost implications, and radiation exposure. 19 This finding was also supported by other studies. 20 21

However, the final decision should be based on the clinical judgment of the physicians. The importance of endoscopy following a difficult tracheostomy tube change in pediatric patients is supported by the limited available data.

Kraft et al. conducted surveys among members of the American Society of Pediatric Otolaryngology (ASPO), revealing that 69% of respondents considered difficult tracheostomy tube changes as a strong indication for endoscopy. 22 Some institutions have adopted routine endoscopic evaluations as a surveillance method for pediatric tracheostomy patients, leading to the identification of a high prevalence of early abnormal airway changes, reaching up to 86.6%. 23

A retrospective chart review was performed on all children with tracheostomies, revealing that 55% exhibited airway abnormalities, including suprastomal granulation (40%), suprastomal collapse (15%), peristomal granulation (10%), and distal tracheal granulation (5%). 24 Among the patients who underwent endoscopy, 48% experienced symptoms, with the most frequent being difficulties during tracheostomy tube changes, ventilation abnormalities, and bloody secretions. 24

Our study has several limitations. It relies on self-reported data, which may be subject to bias. The questionnaire might not cover all possible scenarios of tracheostomy complications, and variations in individual training and institutional protocols might influence responses. Additionally, nonresponse and selection bias are concerns, as healthcare professionals more experienced or interested in tracheostomy care could have been more likely to participate.

## Conclusion

Data regarding the knowledge of healthcare providers in the management of pediatric tracheostomy complications are limited in literature. Our findings reveal significant knowledge gaps among healthcare professionals in managing tracheostomy-related complications in the pediatric population. To address these gaps and enhance patient care, regular training is strongly recommended for healthcare professionals responsible for pediatrics with tracheostomy tube. This training should encompass routine and emergency tracheostomy management, with a specific focus on the most common tracheostomy problems encountered in the age group studied here.

Several studies have emphasized the substantial advantages of incorporating tracheostomy care education modules for both physicians and nurses. 6 13 These educational programs resulted in significant increases in knowledge and confidence levels among healthcare professionals.

Future studies should be conducted systematically or repeated after appropriate education to further investigate and improve tracheostomy care outcomes in the pediatric population.
