# Supplementary material for: Assessment of Healthcare Providers' Knowledge on the Management of Pediatric Tracheostomy Emergencies
Source: Int Arch Otorhinolaryngol. 2025 Apr 28;29(2):1–9. doi: 10.1055/s-0044-1791258 (PMC12037218; doi:10.1055/s-0044-1791258)
Supplement: Supplementary file 1 — Supplementary Material [file 10-1055-s-0044-1791258-s2024051775or.pdf]

Supplementary Material S1 Assessment of Healthcare Professionals' Knowledge of Managing Emergency Tracheostomy Complications in Pediatrics

1. **Level of experience with Tracheostomy Practice in pediatrics:**
   a. Less than 5 years
   b. More than 5 years
   c. Other:
2. **How many times have you been practicing tracheostomy care in pediatrics?**
   a. 1–5 times per week
   b. 1–5 times per month
   c. 6–10 times per month
   d. More than 10 times per month
   e. Few times per year
   f. Never
3. **Have you ever dealt with tracheostomy complications in pediatric patients?**
   a. Yes
   b. No
4. **What is the most common acute complication of tracheostomy in pediatric patients that you know post-operatively?**
   a. Bleeding
   b. False Course of the Cannula
   c. Cannula Obstruction
   d. Pneumomediastinum
   e. Tracheal Spasm
   f. Accidental Cannula Loss
   g. Aspiration Bronchopneumonia
   h. Pneumothorax
   i. Mucus Plug
5. **Do you feel confident to re-insert the tracheostomy tube in cases of tube dislodgment? If you are not confident, choose (0). If confident, choose your confidence level on a scale from 1 to 10**

   0 1 2 3 4 5 6 7 8 9 10
6. **Do you know what is the traction (stay) sutures are?**
   a. Yes
   b. No
7. **What are the main functions of traction (stay) sutures in the emergency setting?**
   a. Traction on these sutures can permit rapid decannulation
   b. To make the change of tracheostomy easier
   c. To stabilize the tracheostomy tube in position
   d. I do not know
8. **Do you know what to do with the introducer/obturator?**
   a. Use upon re-inserting the tracheostomy tube to prevent false passage
   b. Enable straightforward introduction of the tracheostomy tube
   c. Both above answers
   d. I don't know
9. **Answer the following questions based on the given clinical scenario:**

1. **An ICU pediatric patient, day 2 post-surgical tracheostomy without stay sutures, experiences forceful cough leading to accidental dislodgement of the tracheostomy tube. What is the first thing you will do in such a situation?**
   a. Use bag-mask ventilation with jaw-thrust and chin lift
   b. Re-establish the airway by Endotracheal Tube
   c. Try to re-insert the same tracheostomy tube or a smaller tube
   d. Use tracheal dilator as an aid to re-insert the tracheostomy tube
   e. I do not know
2. **In case of sudden massive tracheostomy bleeding, what will you do?**
   a. Clear the airway from blood clots (suction)
   b. I would give tranexamic acid 1 g IV
   c. Hyperinflate tracheostomy tube cuff, apply digital pressure to the base of the neck, and shift the patient to OR
   d. I don't know
3. **In cases where dislodgement of the tracheostomy tube happened and recannulation is impossible, and intubation is difficult, what will you do to secure the airway in an emergency setting?**
   a. Endotracheal intubation using rapid sequence intubation (RSI)
   b. Use supraglottic airway devices using Laryngeal mask airway
   c. Use fiberoptic nasotracheal intubation
   d. Use a surgical blade to widen the tracheostomy site
   e. I don't know

10. **Would you use a fiberoptic scope to examine the airway in case of post-difficult insertion of the tracheostomy tube?**
    a. Always
    b. Sometimes
    c. Rarely
    d. Never
    e. I don't know

11. **Do you think it is important to do neck and chest X-rays after tracheostomy change?**
    a. Yes, in case of an emergency situation only
    b. Yes, during routine change only
    c. Yes, in both routine and emergency
    d. Never
    e. I don't know

 © 2025. The Author(s).
